# Rift Valley Fever Virus Circulating among Ruminants, Mosquitoes and Humans in the Central African Republic

**DOI:** 10.1371/journal.pntd.0005082

**Published:** 2016-10-19

**Authors:** Emmanuel Nakouné, Basile Kamgang, Nicolas Berthet, Alexandre Manirakiza, Mirdad Kazanji

**Affiliations:** 1 Laboratoire de Virologie, Institut Pasteur de Bangui, Bangui, Central African Republic; 2 Research Unit Liverpool School of Tropical Medicine, Organisation de Coordination pour la lutte contre les Endémies en Afrique Centrale, Yaoundé, Cameroon; 3 Department of Zoonosis and Emerging Diseases, Centre International Recherches Médicales de Franceville Gabon, Franceville, Gabon; 4 Institut Pasteur de la Guyane, Cayenne, French Guiana; The Kenya Medical Research Institute (KEMRI), KENYA

## Abstract

**Background:**

Rift Valley fever virus (RVFV) causes a viral zoonosis, with discontinuous epizootics and sporadic epidemics, essentially in East Africa. Infection with this virus causes severe illness and abortion in sheep, goats, and cattle as well as other domestic animals. Humans can also be exposed through close contact with infectious tissues or by bites from infected mosquitoes, primarily of the *Aedes* and *Culex* genuses. Although the cycle of RVFV infection in savannah regions is well documented, its distribution in forest areas in central Africa has been poorly investigated.

**Methodology/Principal Findings:**

To evaluate current circulation of RVFV among livestock and humans living in the Central African Republic (CAR), blood samples were collected from sheep, cattle, and goats and from people at risk, such as stock breeders and workers in slaughterhouses and livestock markets. The samples were tested for anti-RVFV immunoglobulin M (IgM) and immunoglobulin G (IgG) antibodies. We also sequenced the complete genomes of two local strains, one isolated in 1969 from mosquitoes and one isolated in 1985 from humans living in forested areas. The 1271 animals sampled comprised 727 cattle, 325 sheep, and 219 goats at three sites. The overall seroprevalence of anti-RVFV IgM antibodies was 1.9% and that of IgG antibodies was 8.6%. IgM antibodies were found only during the rainy season, but the frequency of IgG antibodies did not differ significantly by season. No evidence of recent RVFV infection was found in 335 people considered at risk; however, 16.7% had evidence of past infection. Comparison of the nucleotide sequences of the strains isolated in the CAR with those isolated in other African countries showed that they belonged to the East/Central African cluster.

**Conclusion and significance:**

This study confirms current circulation of RVFV in CAR. Further studies are needed to determine the potential vectors involved and the virus reservoirs.

## Author Summary

Rift valley fever virus (RVFV) is an arthropod-borne virus that causes serious illness in both animals and humans. RVFV is transmitted by direct contact with infectious tissues or by the bites of infected mosquito species of the *Aedes* and *Culex* genuses. Its distribution in tropical forests in central Africa is poorly documented. We assessed the current circulation of RVFV among livestock and humans in the Central African Republic (CAR) by detecting anti-RVFV immunoglobulin M (IgM) and immunoglobulin G (IgG) antibodies in sheep, cattle and goats and in people living in Bangui who were considered at risk. We also sequenced the complete genomes of two local strains, one isolated in 1969 from mosquitoes and one isolated in 1985 from humans living in forested areas. Sheep were the most frequently infected ruminants. IgM antibodies were found only during the rainy season; the frequency of IgG antibodies did not differ according to season. No evidence of recent RVFV infection was found in humans at risk; however, 16.7% had evidence of past infection. Phylogenetic analysis showed a perfect match of CAR strains with the East/Central African cluster. Our results confirm current circulation of RVFV in CAR. Further studies should be conducted to determine the vectors involved and the virus reservoirs.

## Introduction

Rift Valley fever (RVF) is a viral zoonosis that affects mainly animals but is also found in humans. It is caused by an RNA virus of the *Phlebovirus* genus (Bunyaviridae family), the genome consisting of three RNA segments: large, medium, and small [1,2]. RVFV is transmitted mainly by infected mosquitoes of the *Aedes* and *Culex* genuses, but humans can be contaminated by direct contact with blood (e.g. aerosols, absorption) or tissues (e.g. placenta of stillborns from infected animals) [3,4].

The virus was first identified in 1930 during an epidemic that caused deaths and sudden abortions among sheep on the shores of Lake Naivasha in the Great Rift Valley in Kenya [5,6]. Since then, the virus has spread to most African countries. The disease occurs in endemic and epidemic forms along the east and south coasts of Africa, in West Africa, in Madagascar [7,8] and as far north as Egypt, with a recent outbreak in the Arabian Peninsula [9,10]. Severe episodes of RVF have been reported among humans and animals in southern Africa [11,12,13,14]. The animals most frequently infected are sheep and cattle, followed by goats, with heavy economic losses due to abortions and high mortality rates among juvenile animals [15,16]. RVFV antibodies have been detected in many wild animal species, including ungulates in Kenya [17,18], bats in Guinea [19], and small vertebrates in Senegal and South Africa [20,21]; however, their role in maintenance of the virus in the ecosystem during inter-epidemic periods and their contribution to amplifying outbreaks remain unknown.

RVFV was first isolated in the Central African Republic (CAR) in 1969 from a pool of *Mansonia africana* mosquitoes [22]. It was identified as the causative virus of RVF in 1983 [23,24]. RVFV-specific antibodies have since been detected in humans, and 15 strains of RVFV have been isolated from humans and sylvatic mosquitoes in CAR [25,26], although no RVF outbreak has been reported. Current circulation of RVFV in the CAR is unknown, after a gap of two decades without surveillance; however, as animal breeding plays a large part in the economy of the CAR, an epidemic involving humans and animals is possible.

We undertook a study to assess the current circulation of RVFV in livestock and humans in the CAR. We also sequenced the genomes of local strains isolated in 1969 from wild mosquitoes and in 1985 from humans in a forested area of the country in order to determine the genetic diversity of these strains which will serve as reference for the future.

## Materials and Methods

We performed a prospective cross-sectional study in two phases between November 2010 and November 2012.

### Ethics statement

The national ethical and scientific committees in charge of validating study design in the CAR approved the study design (No. 9/UB/FACSS/CSCVPRE/13). The study was described orally before blood samples were collected from human participants, and participants were included only if they gave written consent; for participants aged ≤ 18 years, a parent or guardian provided written informed consent. The informed consent form included a clause permitting use of the participants’ biological specimens for future research. No endangered or sheltered animal species were used in the survey. Verbal consent for testing their animals was acquired from farmers after the objectives of the study had been explained. Once permission was obtained for blood sample collection, an experienced veterinarian bled the animals gently.

### Sample collection

**Fig 1:**
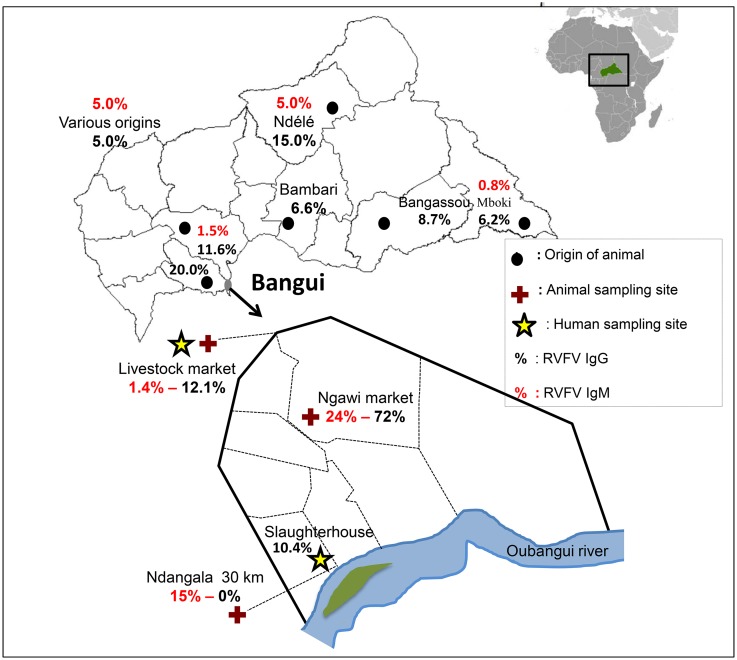
Origin of animals and sampling sites.

Samples were taken during the dry season in the first year and at the same sites during the rainy season in the second year. Blood was collected from sheep, cattle, and goats at three localities: the livestock market situated 13 km north of Bangui for cattle, the Ngawi market in Bangui commercial centre, and Ndangala village located 30 km south of Bangui for sheep and goats (Fig 1). The sex and age of each animal were noted. All animals under 3 years of age were considered juveniles and those over 3 years as adults. Blood samples were also collected from people at risk, such as stock breeders and people working in slaughterhouses and livestock markets. From both animals and humans, venous blood samples were collected in 5-mL Vacutainer tubes (Becton Dickinson, Franklin Lakes, New Jersey, USA), which were placed in a cooler, transported to the laboratory, and centrifuged at 2–8°C for 10 min at 2000 rpm. Each serum sample was separated on collection into two aliquots and stored at –20°C until analysis.

Each person who agreed to participate in this study completed an anonymous questionnaire that included demographics.

The mosquitoes were collected in sylvan environments in 1969, identified, and grouped into pools of up to 30 individuals per species per site; samples were stored at –20°C for a maximum of 4 days in the field, transported to the Institut Pasteur in Bangui, and stored at –80°C until virus isolation. The virus was isolated and amplified by four serial passages in suckling mice brain, as described by Saluzzo and colleagues [27]. The brain suspensions were then lyophilized and stored in sealed glass vials at room temperature until use.

### Serological analysis

Serum samples were analysed for the presence of immunoglobulin M (IgM) and immunoglobulin G (IgG) antibodies to RVF with a SPU-02 RVF IgM and IgG Biological Diagnostic Supplies Ltd (ELISA) kit according to the manufacturer’s instructions. Briefly, plates were coated with a recombinant nucleocapsid RVFV antigen diluted 1:1000 in sodium bicarbonate buffer (pH = 9.6), covered with plate seals and incubated at 4°C overnight. Unbound antigen was removed by washing three times for 15 s each with PBS-T. Plates were then blocked with 10% skimmed milk in PBS (PBS-SM) at 37°C for 1 h and then washed. Test sera were added in duplicate at a dilution of 1:400 in 2% PBS-SM and incubated for 1 h at 37°C. The plates were washed once more, and HRP-conjugated anti-human IgG antibody, diluted 1:25 000 in 2% PBS-SM, was added to each well and incubated for 1 h at 37°C. After a final wash, chromagenic detection of HRP and absorbance measurement were performed as described previously. Negative and positive control sera were included for each plate. Sera samples were considered positive if their calculated optical density (OD) was ≥ 0.29 (net OD serum/net mean OD positive control).

### Genetic analysis

RVFV was isolated from three samples: two strains isolated in 1969 from mosquitoes (ArB1986 and HB74P59) and one from human serum in 1985 (HB1752). It was amplified by inoculation into the brains of newborn mice in a laboratory of biosafety level 3. A brain suspension was prepared, lyophilized, and stored in glass vials at room temperature, and viral RNA was extracted with a QIAmp viral RNA Mini kit (Qiagen, Valencia, California, USA) according to the manufacturer’s instructions [28]. The extracted RNA was treated with Turbo DNase (Life Technologies Inc., Carlbad, California, USA) and then retro-transcribed into cDNA with a SuperScript III First Strand Synthesis kit in the presence of random hexamers. The cDNA generated was amplified with Phi29 enzyme as described previously [29]. A fixed amount of amplified DNA was sequenced in an Illumina Hi-seq 2000 sequencer. An average of 30 × 10^6^ single reads with 100 bases was obtained for each sample [28]. The quality of reads was assessed by FastQC, and the sequences were selected according to their quality. All reads corresponding to the mouse genome sequence were filtered by mapping with Bowtie 2.0 software on a *Mus musculus* Mn10 sequence. The viral reads corresponding to the RVFV genome were selected by a similarity approach with BLASTN search tools [30]. All selected viral reads were assembled with Ray software, with *k* = 25, to obtain the full-length viral genomes [28].

In order to validate our approach for obtaining the complete sequence of RVFV by high-throughput sequencing, the RNA was extracted from three viral strains (HB74P59, HB1752, and ArB1986), and fragments of the small, medium, and large segments were amplified and sequenced. The sequence obtained from HB74P59 was compared with those obtained previously by Bird *et al*. in 2007 [31]; no difference was found in three segments obtained by high-throughput sequencing and classical Sanger sequencing. Moreover, no difference was found in the three segments of strain ArB1986 isolated from an *Aedes palpalis* mosquito and that of a strain isolated in the same city, Loko-Zinga, in 1969 but from another arthropod, *Mansonia africana*.

### Statistics

The distribution of serological results for RVFV was analysed by species, season, and human age and sex and presented as proportions. The effect of each variables on the RVFV positivity was examined using the chi-squared test. *P* values < 0.05 were considered statistically significant. The variables were then put in a Logistic regression model in stepwise manner. The likelihood ratio test was used to compare the model with and without the variable. In case there was no evidence that the variable fitted, it was dropped (parsimonious model). Statistical analyses were performed with STATA software version 11.

## Results

### Animal samples

A total of 1271 animals were sampled, comprising 727 cattle, 325 sheep, and 219 goats (Table 1). The overall seroprevalence in animals was 1.9% for anti-RVFV IgM antibodies and 8.6% for IgG antibodies. The seroprevalence varied significantly by ruminant. The IgM antibody titre, which indicates recent circulation of RVFV, was 4.3% in sheep, 1.4% in goats, and 1.1% in cattle (*P* < 0.0001), whereas the IgG seroprevalence was 12.9% in sheep, 7.8% in cattle, and 5.0% in goats (Table 2). However, the multivariate analysis showed that IgM and IgG seropositivity rates in sheep were higher than other species (OR = 1.8, 95% CI = 1.1–3.0) (Table 1). No significant difference in seropositivity was found between male and female animals (Table 3). The IgG seropositivity rate was 9.6% in adults and 5.1% in juveniles (*P* < 0.02), but no significant difference in positivity for anti-RVFV IgM antibodies was found between juveniles and adults (Table 3). The IgM and IgG seropositivity rates varied significantly according to the origin of the sample. No animals positive for IgG antibodies were found in Ndangala, whereas most of those positive for IgM antibodies were originated from this site, and cattle market are less likely to be IgM positive (OR = 0.1, 95% CI = 0.0–0.5) (Tables 1 and 3). All animals with positive IgM were found in the rainy season, and IgG seropositivity was more pronounced during dry season (Table 3). None of the livestock owners reported cases of abortion or death in the months before sampling that would indicate RVFV infection in their herds.

**Table 1:** Multivariate analysis of risk factors for RVF virus seropositivity in animals.

	No.	Positive IgM					Positive IgG				
		No.	%	*P*	Multivariate analysis		No.	%	*P*	Multivariate analysis	
					OR	95% CI				OR	95% CI
Species											
Cattle	727	8	1.1	0.002	Reference		57	7.8	0.003	Reference	
Goat	219	3	1.4		0.3	0.0–2.2	11	5.0		0.8	0.4–1.7
Sheep	325	14	4.3		0.9	0.2–4.7	42	12.9		1.8	1.1–3.0
Sex											
Male	551	6	1.1	0.07	-		39	7.1	0.204	-	
Female	719	19	2.6		-		71	10.0		-	
Age											
Juvenile	273	3	1.1	0.36	-		14	5.1	0.019	-	
Adult	998	22	2.2		-		96	9.6		-	
Origin											
Ndangala	69	6	8.7	< 0.0001	Reference		0	0.0	< 0.0001	Reference	
Ngawi	119	9	7.6		0.7	0.2–2.4	22	18.5		0.5	0.8–2.8
Cattle market	1083	10	2.0		0.1	0.0–0.5	88	8.1		N/A	
Season											
Rainy	732	25	3.4		NA		88	12.0	< 0.001	Reference	
Dry	539	0	0.0	< 0.001	NA		22	2.1		0.3	0.1–0.5

NA, not applicable; OR, odd ratio; CI, confidence interval; IgM, Immunoglobulin M; IgG, Immunoglobulin

**Table 2:** Serological results for RVFV by species and season in the CAR.

	No.	Positive IgM			Positive IgG		
		No.	%	*P*	No.	%	*P*
Species							
Human	335	0	0.0		56	16.7	
Cattle	727	8	1.1	0.002*	57	7.8	0.003
Goat	219	3	1.4		11	5.0	
Sheep	325	14	4.3		42	12.9	
Season*							
Dry	539	0	0.0	<0.001	22	2.1	<0.001
Rainy	732	25	3.4		88	12.0	

IgM, Immunoglobulin M; IgG, Immunoglobulin G; No., number positive

*For animals only

**Table 3:** Prevalence of RVFV in ruminants.

	No.	Positive IgM			Positive IgG		
		No.	%	*P*	No.	%	*P*
Sex							
Male	551	6	1.0	0.07	39	7.1	0.09
Female	719	19	2.6		71	10.0	
Age							
Juvenile	273	3	1.1	0.36	14	5.1	0.03
Adult	998	22	2.2		96	9.6	
Origin							
Ndangala	69	6	8.7	0.001	0	0	0.001
Gawi	119	9	7.6		22	18.5	
Cattle market	1083	10	2.0		88	8.1	

IgM, Immunoglobulin M; IgG, Immunoglobulin G

### Human samples

Blood samples were collected from 335 people who were regularly in contact with blood from the animals. The mean age (±SD) was 36.3 years (±18.1), and the sex ratio (M/F) was 6.0/1 (287/48). No evidence of recent RVFV infection (absence of IgM) was found in human samples; however, 16.7% had evidence of past infection (IgG alone). Of these, 7.7% were stock breeders, 6.6% were butchers, 1.5% were slaughterhouse workers, and 0.9% were veterinarians (Table 4). A higher positivity rate was observed among people over 25 years of age (*P* = 0.04), and 17.8% (51/287) of males and 10.4% (5/48) of females were positive for IgG (*P* = 0.29) (Table 4).

**Table 4:** Prevalence of RVFV in people at risk.

		Positive IgG		
Characteristic	No.	No.	%	*P*
Sex				
Male	287	51	17.8	0.29
Female	48	5	10.4	
Age (years)				
< 15	23	0	0.0	0.04
15–24	39	4	10.4	
25–34	90	18	20.0	
35–44	88	21	23.9	
> 44	95	13	13.7	
Trade*				
Veterinarian	28	3	10.7	0.72
Slaughterhouse worker	41	5	12.2	
Butcher	41	9	22.0	
Breeder	77	10	13.0	

IgG, Immunoglobulin G; N, number positive

* Differences between groups are due to the missing data

### Genetic analysis of complete genome isolated from mosquitoes

**Fig 2:**
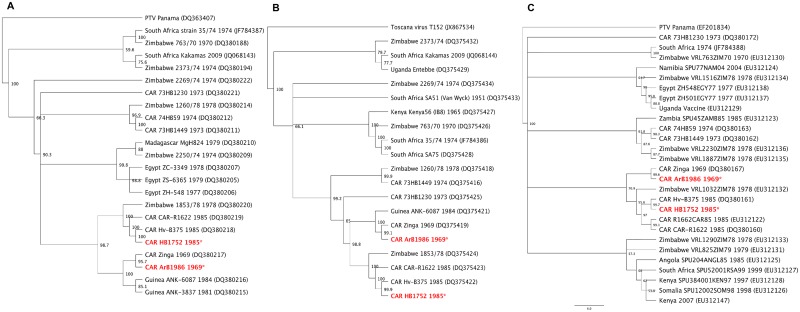
Phylogenetic tree of selected RVFV segments: (A) small (S), (B) medium (M) and (C) large (L). Analysis at nucleic acid level with sequences available in GenBank. The tree was generated by the neighbor-joining method with Geneious software for Mac (Geneious version 6.1 created by Biomatters) by the boostrapping approach with 1000 replicates. Values are showed as percentages.

A third RVFV strain obtained by high-throughput sequencing was isolated in Bangui at the end of December 1984 (HB1752). Genomic analysis of three segments showed that it was identical to a strain isolated in Bangui 3 months earlier; however, RVFV strains isolated from vectors in 1969 and from human cases in the CAR several decades later had different nucleic sequences, even though they belonged to the same cluster (Fig 2). The complete genome sequence was made available to GenBank (HB1752 strain small, medium, and large accession numbers KJ782452, KJ782453, and KJ782454 respectively; ArB1986 strain small, medium, and large accession numbers KJ782455, KJ782456, and KJ782457, respectively).

## Discussion

This study, the first in the CAR since RVFV was isolated in 1985, shows that the virus continues to circulate in central Africa. The overall prevalence in animals in this study was lower than that reported in Comoros in 2009 (39% in sheep and 33.5% in goats) [32], in Madagascar in 2008 (24.7% in small ruminants) [33], and in Mozambique (35.8% in sheep and 21.2% in goats) [34]. The same ELISA kits were used in all these studies, suggesting that the differences are due to climatic factors, entomological parameters, agro-ecological conditions, or sampling strategies.

The higher prevalence in sheep is consistent with previous work, indicating that this species is preferentially infected with RVFV [15]. Most infected animals, especially sheep, were found in Ndangala, a rural forested area south of Bangui that has more rainfall than the rest of the country, with lower temperatures and constant humidity in the rainy season, during which time there is little husbandry. The high prevalence observed at this site, with the presence of IgM antibodies, suggests endemic virus circulation, which would be maintained by a sylvatic cycle involving wild animals and mosquitoes, as suggested by Olive *et al*. [35]. In a previous study in a forested area of the CAR, RVFV was isolated from wild mosquitoes, including *Ae*. *palpalis* [26]. In the rainy season, there are many potential breeding sites, which increases the density of vectors and subsequently increases transmission of arboviral diseases such as RVFV. IgM, which indicates recent infection, was present only during the rainy season, but IgG was also significantly associated with the rainy season. These finding are consistent with those in Mauritania and Senegal that indicate that the risk factors for RVF are linked to heavy rainfall and the presence of large temporary masses of surface water [36,37]. In a previous study, seropositivity for RVFV was associated with increased numbers of mosquito vectors [38]. Although we did not conduct entomological surveys, recent entomological surveillance for yellow fever identified several species of *Aedes* mosquito, including *Ae*. *cuminsii*, *Ae*. *circumluteolus*, and *Ae*. *palpalis* [39], which are known vectors of RVFV [40].

The seroprevalence of RVFV was higher in adult than in juvenile animals. Similar results were reported in Mauritania and Senegal [32,33], supporting the hypothesis of endemic circulation of the virus, as older animals would have longer exposure than younger ones [34]. The presence of IgG in young animals (< 3 years) in this study suggests recent circulation of the virus. This result is compatible with the IgM titres in each species, with high titres in samples taken from sheep in Ndangala (Table 2). The absence of IgG in animals from this region indicates that introduction of the virus south of Bangui is recent. Recent introduction of the virus associated with environmental modifications such as deforestation, population displacement due to the socio-political crisis, and introduction of a new vector competent for RVFV [41] could increase the risk for emergence of an epidemic.

As no epidemic of RVF or obvious clinical signs of the disease (such as abortions) was observed, the infections were minor or sub-clinical [34]. In a study in Madagascar, circulation of RVFV during the dry season did not result in clinical cases [33]. Viral activity may be maintained in mosquitoes near rivers that do not dry up during the dry season, resulting in a low level of transmission among domestic animals. These observations and the recent epidemics in East Africa illustrate the risk for introduction of pathogenic strains of RVFV to CAR from countries such as South Sudan, which shares a long border with CAR and has had many epidemics and epizootics of RVF.

The absence of anti-RVFV IgM antibodies among people regularly exposed to animals would appear to indicate that contact with the virus is uncommon and the public health risk is low. Nevertheless, the presence of IgG among breeders and butchers, who are in contact with the blood of these animals, should alert the authorities to strengthen surveillance of circulation of this virus. Studies to isolate the virus in the vector (mosquitoes) and longitudinal studies in sheep, goats, and cattle should be conducted to detect clinical cases, particularly among sheep and herders in Ndangala village, where evidence of current circulation of the virus was found. As most of the inhabitants of the rural areas in which the small ruminants were sampled are farmers who share the same environment as their animals and may have the same exposure to mosquitoes, a study should also be carried out to determine the prevalence of RVFV antibodies and to establish whether RVF occurs regularly in these zones and is thus a neglected cause of morbidity and mortality.

Our study was limited to Bangui and neighbouring areas because animals are brought to the capital from all regions of the country. As we were unable to isolate recent strains of the virus, we could not establish the precise geographical origin of the viral strains currently circulating in the CAR. Nevertheless, on the basis of genomic data for old strains, the same strain may be circulating relatively freely in several vectors in a defined geographical area over a long period. Furthermore, although IgG responses persist for several years in continually exposed people, we were unable to obtain a second sample at an interval of 2 weeks and therefore could not demonstrate seroconversion, which would support recent infection. The low IgG titres and high IgM titres found in the Ndangala region suggest recent introduction of the virus into this forested area, probably associated with illegal movement of sheep and goats from the Democratic Republic of Congo.

In view of the highly precarious situation in CAR, a large-scale molecular study will not be possible in the short term; furthermore, the socio-political upheaval in the country may change socioeconomic and environmental conditions and the time of infection before the study is conducted.

Comparison of the sequences of strains isolated from vectors and humans in the CAR with those isolated in other African counties show that they belong to the East/Central African cluster, confirming RVFV strain exchanges among geographical areas. Propagation of RVFV from East Africa to other regions was noted in Saudi Arabia and Yemen in 2000–2001 [42] and in Chad in 2001 [43] during previous RVF outbreaks.

## Conclusion

The results of this first study conducted in both humans and animals in the CAR are important for public health. They shows a high prevalence of RVFV in an area where neither epidemics nor clinical cases of RVF have been reported previously. These results are also important because, in the forested area south of Bangui where there is little husbandry, there is nevertheless low virus noise, which might suggest the presence of a reservoir that has come nearer to human habitats. Unexpectedly, we found low IgM titres in regions of previous intensive animal husbandry, because animal density in these areas has fallen sharply due to the migration of herders in response to the continuing instability in the CAR.

Other studies are required to elucidate and measure the environmental risk factors for infection with RVFV in order to predict epidemics, and entomological studies should be performed to identify all the potential vector species to better understand the ecological and climate factors that favor the distribution of RVFV.

## Supporting Information

S1 Checklist STROBE statement for cross-sectional studies. (DOC)
